# Abscopal effect induced by conventional fractionated radiotherapy following anti-PD-1 immunotherapy in pulmonary metastatic thymic squamous cell carcinoma: a case report and literature review

**DOI:** 10.3389/fimmu.2026.1733066

**Published:** 2026-02-18

**Authors:** Minghui Cui, Jie He, Fang Zhang, Yiqian Zhang, Ang Gao, Jie Liu

**Affiliations:** 1 School of Clinical Medicine, Shandong First Medical University, Jinan, China; 2 Department of Oncology, Zhangqiu People’s Hospital, Jinan, China; 3 Department of Oncology, Central Hospital Affiliated to Shandong First Medical University, Jinan, China

**Keywords:** abscopal effect, conventional fractionated radiotherapy, immunotherapy, optimized target volume delineation, thymic squamous cell carcinoma

## Abstract

**Background:**

Radiotherapy (RT) can enhance immune control of distant metastases, known as the abscopal effect (AE), but it doesn’t significantly alter the immunosuppressive tumor microenvironment (TME), resulting in low AE incidence. Combining RT with immunotherapy (especially anti-PD-1/PD-L1 agents) has increased AE occurrences, though questions remain about this approach, particularly in tumors with low immunogenicity such as thymic squamous cell carcinoma (TSCC).

**Case description:**

A 73-year-old woman with advanced TSCC and multiple metastases experienced disease progression after two therapies. Following palliative conventional fractionated radiotherapy (CFRT) (40Gy) for thoracic metastases, her pleural lesions outside the radiation field significantly reduced, indicating an AE. Despite subsequent immunotherapy and antiangiogenic drugs, treatment efficacy was unsatisfactory due to severe lymphopenia, possibly contributing to disease progression.

**Conclusion:**

The rise of immunotherapy challenges traditional RT. To enhance AE occurrence in practice, factors like radiation dose, irradiation site, timing with ICIs, ICI drug choice, patient health, disease stage, and tumor traits must be considered. This case demonstrates that CFRT can induce an AE in TSCC but also highlights the associated risk of severe lymphopenia that may limit its durability. Monitoring and mitigating lymphopenia are crucial in optimizing combined therapy outcomes. This case provides new clinical evidence for treating recurrent TSCC with combined therapy, though more research on its immunological mechanisms is needed.

## 1 Introduction

Recent studies have demonstrated that radiotherapy (RT) not only exerts localized effects by directly eliminating tumor cells but also enhances immune-mediated control of distant metastases through the systemic immune response it induces, a phenomenon known as the abscopal effect (AE) (1). This effect is particularly pronounced in immunogenic tumors (2–4). Research has confirmed that the AE is mediated by the remote activation of immune pathways and the remodeling of the tumor microenvironment (TME) (5). Clinically, common fractionation schedules include conventional fractionation (CFRT) (1.8–2 Gy per fraction), hypofractionation (>2 Gy per fraction), and altered fractionation (e.g., hyperfractionation with smaller doses multiple times daily, and accelerated fractionation to reduce overall treatment time). Consequently, the combination of RT with immunotherapy (iRT) has emerged as a significant focus of research in recent years (6). However, the precise mechanisms and influencing factors of the AE remain inadequately understood. Research indicates that the timing between RT and immunotherapy can affect the immune system’s recovery and remodeling processes, potentially mitigating chronic immunosuppression. The PACIFIC trials highlighted the significance of treatment duration (7). Additionally, research emphasizes the critical role of accurately targeting hypermetabolic lesions and metastatic lymph nodes in achieving the AE (8–16). Factors significantly influencing the occurrence of AE encompass tumor pathology type, RT dosage and fractionation patterns, the integration of various immune checkpoint inhibitors (ICIs), and the sequencing of treatments. We present a case of recurrent thymic squamous cell carcinoma (TSCC) in which AE was successfully induced at a cumulative dose of 40 Gy, after only 20 fractions of CFRT, a scenario not previously documented in this specific context.

## 2 Case description

A 73-year-old female patient with a history of thymic squamous cell carcinoma (TSCC) spanning over a decade, who had previously undergone multiple therapeutic regimens for metastatic disease, presented with worsening chest tightness and dyspnea persisting for one week. She was admitted to our hospital on April 24, 2023. The patient had no history of chronic diseases, infectious diseases, autoimmune disorders, smoking, alcohol consumption, or a family history of cancer. Physical examination revealed a firm, non-tender mass on the right clavicle, and wheezing was auscultated in the lungs. Vital signs were stable, and no abnormalities were detected in other physical examinations.

In 2013, a contrast-enhanced chest computed tomography (CT) scan identified a mass measuring approximately 4.8 × 3.4 cm in the anterior and middle mediastinum, slightly to the right. The mass was characterized by patchy calcification, heterogeneous enhancement, and indistinct boundaries with the pericardium, partially extending into the right lung field. On September 27, 2013, the patient underwent surgical resection of the anterior mediastinal mass and a wedge resection of the right upper lobe under general anesthesia with endotracheal intubation. Postoperative pathological analysis confirmed the diagnosis of TSCC, supported by immunohistochemical findings. The patient subsequently underwent a treatment regimen comprising chemoradiotherapy (CRT) at a total dose of 5400 cGy delivered in 30 fractions, in conjunction with four cycles of chemotherapy involving paclitaxel and lobaplatin. This comprehensive therapeutic approach resulted in an impressive disease-free survival (DFS) period of seven years.

In 2020, the patient self-identified a mass in the right supraclavicular region, which exhibited progressive enlargement. By July 21, 2021, imaging findings revealed multiple enlarged lymph nodes in the superior mediastinum, suggestive of tumor recurrence. Pathological examination confirmed the presence of metastatic poorly differentiated carcinoma. Consequently, the patient was initiated on a first-line treatment regimen consisting of five cycles of chemotherapy with albumin-bound paclitaxel (300 mg on day 1) and nedaplatin (45 mg on days 1 and 2) administered every 21 days. A follow-up assessment on December 23, 2021, indicated disease progression. In response to the patient’s condition, a second-line treatment regimen was initiated on December 23, 2021, and January 13, 2022. This regimen comprised camrelizumab (AiRuiKa^®^) (200 mg) for immunotherapy, administered on both aforementioned dates, in combination with apatinib (250 mg every other day) and tegafur (40 mg twice daily from day 1 to 14). However, the disease was not effectively controlled, and the patient exhibited poor tolerance to treatment. Following this, the patient ceased attending scheduled hospital check-ups and intermittently self-administered apatinib and tegafur.

**Figure 1:**
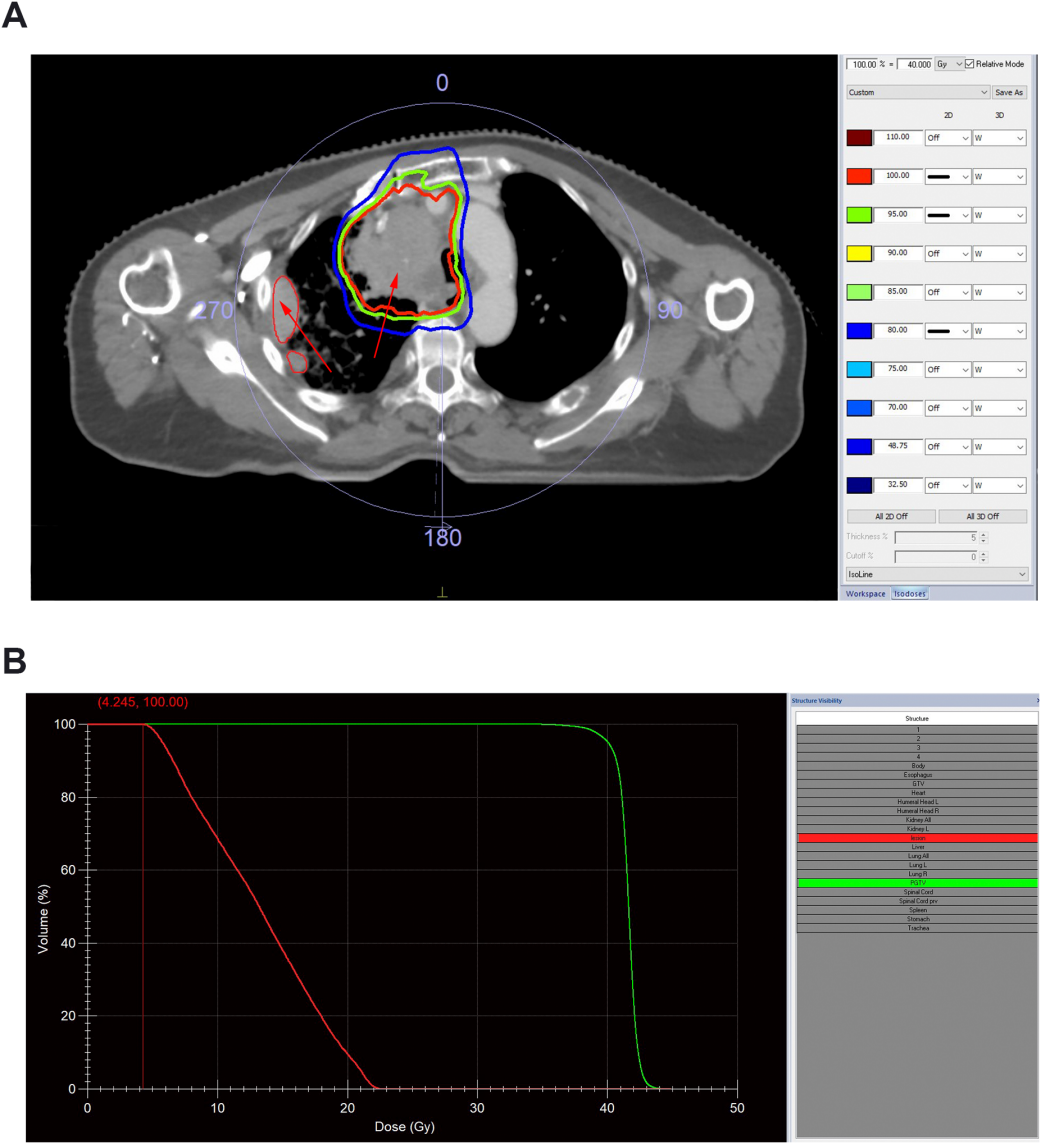
Dosimetric distribution analysis and validation of the pleural lesion outside the primary radiotherapy target volume. **(A)** Axial CT simulation image illustrating the isodose distribution of the primary mediastinal target volume. The bold red, green, and blue lines represent the 100%, 95%, and 80% isodose lines, respectively. The arrows indicate high-dose regions; the pleural lesion is specifically contoured with a thin red line (as indicated by the arrow) and is located entirely outside the 80% isodose line. **(B)** Dose-volume histogram (DVH) analysis of the out-of-field pleural lesion generated by the treatment planning system (TPS). The analysis confirms that the lesion received only low-dose scattered radiation ranging from 1 to 5 Gy, with a calculated potential physical dose of 4.245 Gy to the pleural lesion.

At the current admission, the patient presented with extensive mediastinal lymph node metastases causing dyspnea and superior vena cava syndrome. Given the lack of effective systemic options and declining performance status, palliative CFRT to the thorax was chosen to alleviate symptoms. Radiotherapy commenced on April 10, 2023, targeting the mediastinal lymph nodes (GTV). A 5 mm margin was added to create the PTV, with an initial volume of 201.471 cm³. A dose of 50 Gy was planned using volumetric modulated arc therapy (VMAT). Upon reaching a dose of 40 Gy (May 9, 2023), a repositioning CT scan revealed significant shrinkage not only of the in-field mediastinal lymph nodes but also of a pleural lesion located entirely outside the radiation field (**Figure 1A**). Dose-volume histogram (DVH) analysis confirmed that this out-of-field lesion received only low-dose scattered radiation ranging from 1 to 5 Gy, with a mean calculated dose of 4.245 Gy (**Figure 1B**). Based on this favorable response, the treatment plan was adapted, and the PTV was reduced to 132.180 cm³. The patient successfully completed the planned CFRT course on May 22, 2023, receiving a total dose of 58 Gy in 29 fractions without acute complications.

Following CFRT, the patient initiated a third-line systemic therapy with sintilimab (200 mg on day 1) combined with anlotinib (8 mg orally on days 1-14) every three weeks, starting June 11, 2023. Sintilimab was later discontinued due to suspected immune-related pneumonia, while anlotinib monotherapy was continued.

**Figure 2:**
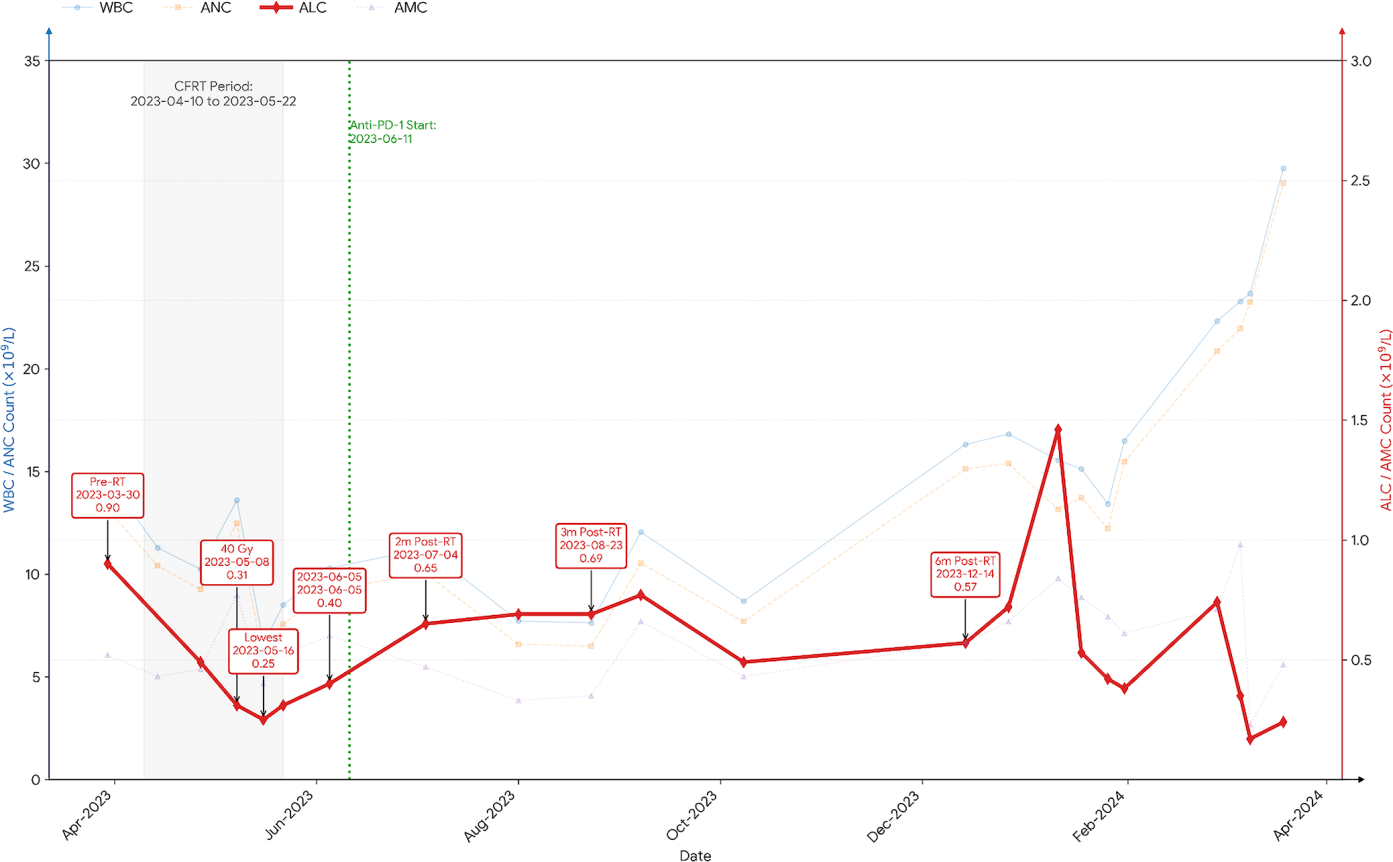
Absolute counts of white blood cells (WBC, blue line), neutrophils (ANC, blue dashed line), lymphocytes (ALC, bold red line), and monocytes (AMC, red dashed line) from March 2023 to March 2024. The left y-axis (blue) corresponds to WBC and ANC values, and the right y-axis (red) corresponds to ALC and AMC values, all expressed as ×10^9^/L. The timeline marks two key interventions: palliative chest radiotherapy (RT, grey shaded area; 10 April to 22 May 2023) and the initiation of Sintilimab (anti-PD-1) therapy (green vertical dotted line; 11 June 2023). Key time points for the ALC trajectory are annotated with dates and absolute counts: pre-RT baseline (0.90, 30 Mar 2023), during RT at 40 Gy (0.31, 8 May 2023), nadir (0.25, 16 May 2023), initial recovery (0.40, 5 Jun 2023), and follow-ups at 2, 3, and 6 months post-RT (0.65, 0.69, 0.57). For clarity, WBC, ANC, and AMC traces are plotted with reduced opacity, and closely spaced annotations are vertically offset. The profile demonstrates treatment-associated lymphodepletion during radiotherapy, followed by subsequent recovery. A marked, concurrent elevation in myeloid cell counts (WBC/ANC) is observed in the late phase of the observation period.

**Figure 3:**
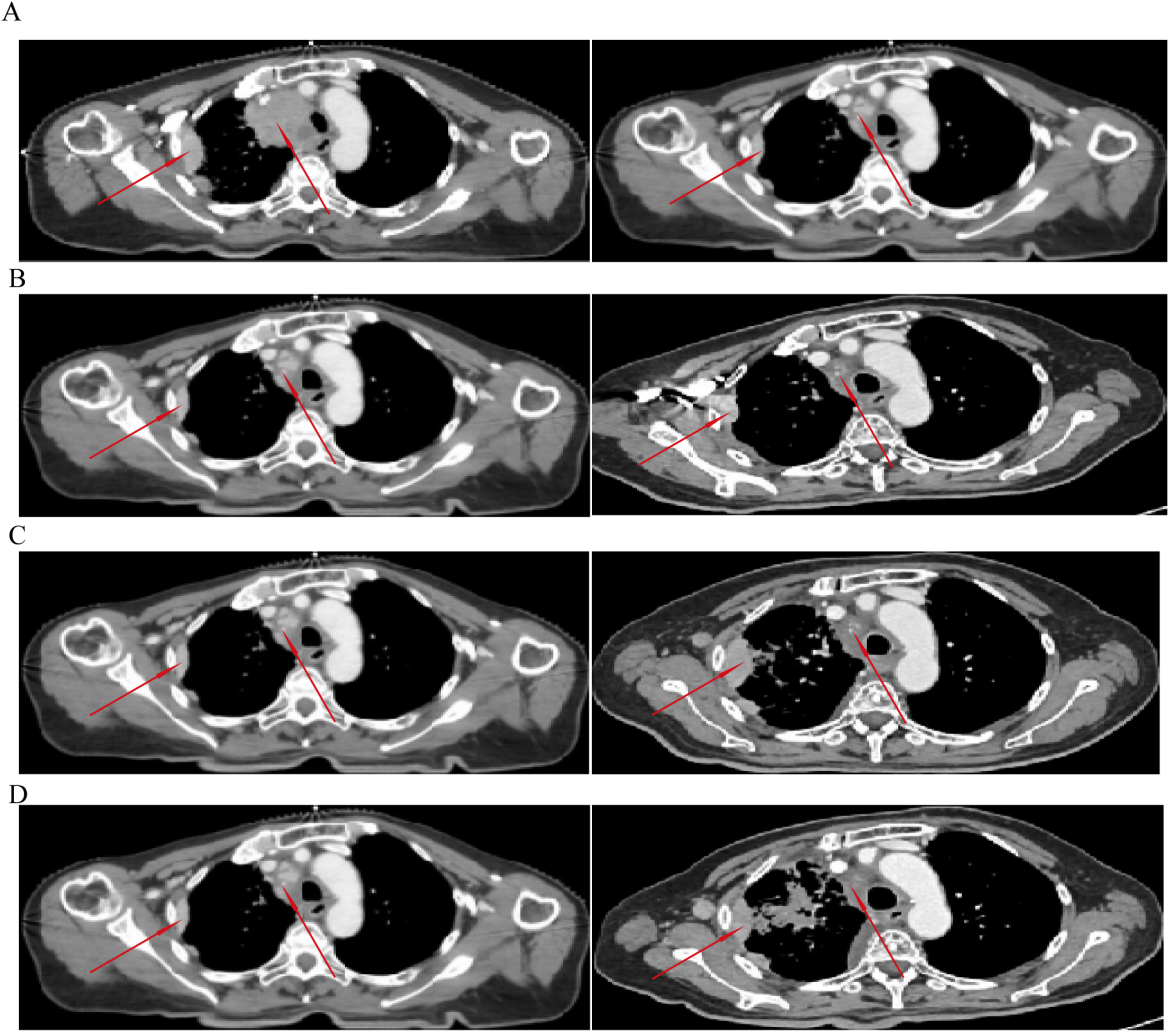
Chronological evolution of primary and abscopal lesions following radiotherapy and subsequent disease progression. **(A)** Imaging comparison between April 6, 2023 (baseline) and May 8, 2023 (radiation therapy simulation). At the time of simulation, both the mediastinal lymph nodes within the target volume and the abscopal pleural lesion outside the target volume showed regression compared to baseline. **(B)** Imaging comparison between May 8, 2023 and June 5, 2023 (1 month post-radiotherapy). The abscopal lesion exhibited continued regression. **(C)** Imaging comparison between May 8, 2023 and July 4, 2023 (2 months post-radiotherapy). The abscopal lesion demonstrated signs of progression. **(D)** Imaging comparison between May 8, 2023 and August 24, 2023 (3 months post-radiotherapy). The abscopal lesion showed further progression, accompanied by broader clinical deterioration including increased right pleural effusion, partial right lung atelectasis, and signs of lymphangitic carcinomatosis. These findings suggest that the clinical course transitioned from an earlier immune-mediated regression phase to a state of disease progression with new-onset complications.

Dynamic changes in peripheral blood cell counts were meticulously monitored throughout the treatment course (**Figure 2**). The baseline absolute lymphocyte count (ALC) before CFRT was 0.9×10^9^/L (March 30, 2023). After the initiation of CFRT, the ALC showed a gradual initial decline. A marked decline was observed when the cumulative dose reached approximately 40 Gy, with the ALC dropping to a nadir of 0.25×10^9^/L on May 16, 2023, indicating the onset of severe radiation-induced lymphopenia (RIL). It is noteworthy that the repositioning scan performed at this same dose point (40 Gy) demonstrated significant regression of both the in-field and out-of-field lesions compared to the baseline (**Figure 3A**). Partial recovery of ALC was observed after radiotherapy completion. The white blood cell (WBC) and absolute neutrophil count (ANC) fluctuated during treatment and showed a marked upward trend from late 2023, coinciding with suspected pneumonitis and eventual disease progression. No systemic corticosteroids were administered prophylactically during radiotherapy.

Serial imaging follow-up delineated the evolutionary course of the lesions (**Figure 3**). Compared to the baseline (April 6, 2023), both the in-target and the out-of-field pleural lesions showed regression at the time of radiotherapy simulation (May 8, 2023) (**Figure 3A**). At one month post-RT (June 5, 2023), the out-of-field lesion continued to regress (**Figure 3B**). However, subsequent scans at two (July 4, 2023) and three months (August 24, 2023) post-RT demonstrated clear progression of the abscopal lesion (**Figures 3C, D**), accompanied by signs of broader disease progression including increased pleural effusion, lung atelectasis, and lymphangitic carcinomatosis, marking a transition from an early phase of potential immune-mediated regression to a progressive disease state.

**Figure 4:**
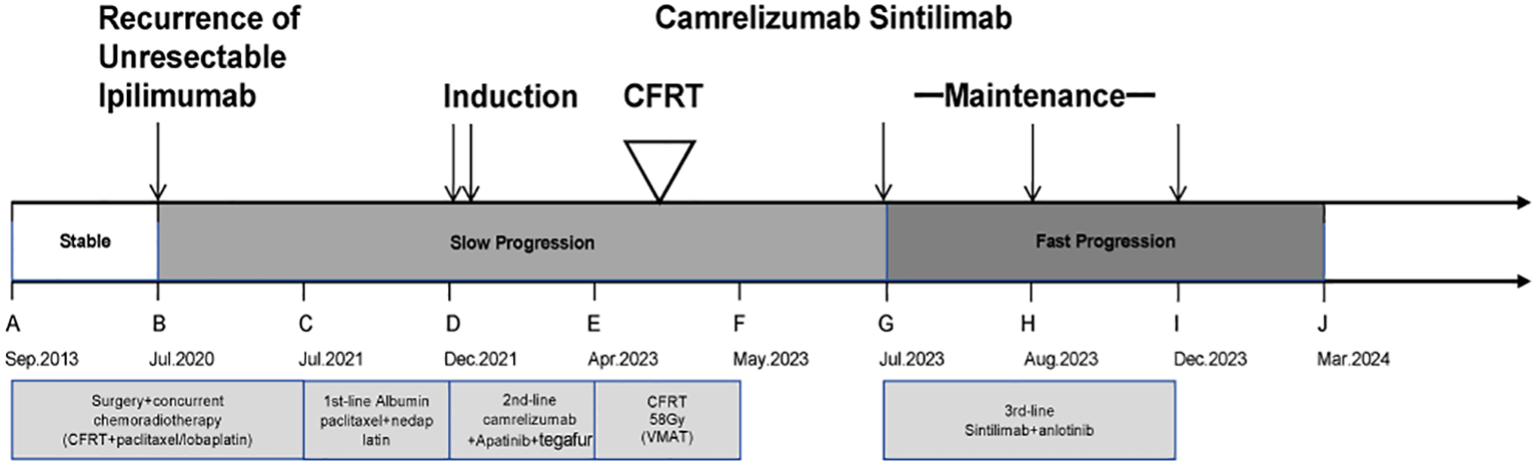
The timeline with relevant data from the episode of care.

By December 15, 2023, CT imaging confirmed extensive systemic disease progression. The patient ultimately succumbed to respiratory and circulatory failure on March 21, 2024. A timeline summarizing the entire episode of care with key data is presented in **Figure 4**.

## 3 Discussion

To date, there have been no reported cases of AE manifesting in patients with recurrent TSCC following the combined application of RT and immunotherapy. Previous studies have primarily reported RT-induced AE in immunogenic tumors, indicating a correlation between AE occurrence and tumor immunogenicity (2–4). JZ et al. have demonstrated a positive correlation between tumor immunogenicity and the occurrence of AE following RT (2–4). Tumors with a high tumor mutational burden (TMB) are characterized by a substantial number of novel antigens and exhibit pronounced immunogenicity (17). According to the study by Milan Radovich et al, Thymoma and thymic cancer have a low TMB, with averages of 0.48 and 1.2 mutations per megabase, respectively (18–20). An 89-year-old patient with metastatic neuroendocrine large cell thymic carcinoma showed regression and even disappearance of lung lesions in non-irradiated areas following palliative RT for a sternal mass. This case is considered the first documented instance of AE associated with thymic carcinoma in the literature (21). Furthermore, a 76-year-old female patient with B3 thymoma and multiple lung metastases demonstrated significant regression of metastatic lesions in non-irradiated areas following local RT, which further confirms the AE induced by RT (22). These cases challenge the conventional understanding of AE triggers. Although thymoma is typically considered an ‘immunologically cold’ tumor with infrequent TMB, RT can effectively activate its anti-tumor immune response, thereby inducing an AE (23). Consequently, future AE strategies should prioritize individualized RT modalities and TME reprogramming over merely focusing on tumor type selection. In this case, the primary target received a high-dose regimen, whereas the regressing non-target lesion was exposed to a mean scatter dose within the LDRT range. This suggests the observed systemic regression likely resulted from a combined mechanism: systemic immune activation triggered by high-dose irradiation coupled with local TME modulation by the incidental low-dose scatter. This low-dose component may have helped mitigate the “donut effect,” where a stromal barrier excludes T-cells from the tumor core. Preclinical evidence indicates very low-dose radiation can create breaches in this barrier, promoting T-cell infiltration (61). Consequently, this case highlights that when designing combination therapies aiming to harness RT as an *in situ* vaccine, it is necessary to consider not only the dose to the primary target but also the potential immunomodulatory impact of low-dose exposures to overcome immune exclusion and optimize therapeutic outcomes.

RT has the potential to modify the tumor immune microenvironment, potentially converting “cold tumors” into “hot tumors.” However, it is insufficient to fundamentally alter the immunosuppressive nature of the TME, resulting in a low incidence of RT-induced AE (24, 25). With the advent of immunotherapy, the combination of RT and immunotherapy has been observed to increase the occurrence of AE (2, 6). Despite this progress, several critical questions remain unanswered in this combined modality therapy. These include the optimal selection of RT technology, the appropriate fractionation scheme, the total radiation dose, the specific sites for RT, the timing interval between RT and immunotherapy, and the sequencing of these treatment modalities. Hypofractionated radiotherapy (HFRT), characterized by doses exceeding 2.5 Gy per fraction, is recognized for promoting the AE by rapidly releasing tumor-associated antigens and damage-associated molecular patterns (DAMPs). This process activates dendritic cells through TLR4/NF-κB signaling pathways and enhances systemic CD8+ T-cell immunity (26, 27). Clinical evidence substantiates these findings, demonstrating that carbon-ion RT administered at 12−16 Gy per fraction effectively induces AE in thymic carcinoma, while stereotactic body radiotherapy (SBRT) at 18 Gy in a single fraction, when combined with anti-PD-1 therapy, enhances survival outcomes in patients with non-small cell lung cancer (NSCLC) brain metastases (28). However, the successful induction of an AE using CFRT (2 Gy per fraction) in recurrent thymic squamous cell carcinoma (TSCC), as reported here, remains a notable and less common clinical observation. Nevertheless, the dose-fractionation schedule is critically important, as ultra-high doses (≥15 Gy per fraction), a category of HFRT, may entail dual immunosuppressive risks. Such high doses can facilitate the recruitment of immunosuppressive CD4+FoxP3+ regulatory T cells (Tregs) through the upregulation of transforming growth factor-beta (TGF-β) and activate Trex1, which degrades cytosolic deoxyribonucleic acid (DNA), thereby inhibiting the cyclic GMP-AMP synthase-stimulator of interferon genes (cGAS-STING) pathway (29, 30). Concurrently, these doses may induce type I interferon (IFN-I) stimulates the secretion of amphiregulin, leading to the polarization of myeloid cells towards an immunosuppressive phenotype and subsequently accelerating disease progression (31). LDRT, with doses ranging from 0.5 to 2.5 Gy per fraction, is hypothesized to modulate the TME and stimulate immune responses (32–34). A dose comparison study conducted by Yin and Herrera et al. demonstrated that single fractions of 2 Gy and 1 Gy significantly enhanced immune cell infiltration (35, 36). This indicates that the activation of immune responses by RT is dose-dependent within a specific range, with higher single doses (≥15 Gy) potentially exacerbating immunosuppressive effects (29, 30, 37). Nonetheless, the immunogenic potential of CFRT (daily dose ≤2 Gy) remains less well understood.

A critical observation in this case was the onset of AE at a cumulative dose of 40 Gy, after only 20 fractions of CFRT, significantly preceding the completion of treatment. This finding challenges the traditional understanding that AE typically emerges months following the conclusion of RT, with reported onset ranging from 0.5 to 24 months, and is more commonly associated with high-dose or hypofractionated regimens (38). We hypothesize that CFRT, administered at a daily dose of 2 Gy, can reach a critical threshold for the release of tumor antigens and the activation of dendritic cells (DCs) at cumulative doses ranging from 20 to 40 Gy (35). Activated DCs play a vital role in linking innate and adaptive immunity by processing and presenting tumor antigens to T cells, thereby facilitating the generation of tumor-specific cytotoxic T lymphocytes (CTLs) (39). Notably, a cumulative dose of 20−40 Gy has been shown to optimize the balance between tumor cell eradication and immune activation, promoting sufficient antigen release without causing excessive immunosuppression or lymphopenia (40). Our patient developed profound lymphopenia (0.25 × 10^9^/L). This observation critically highlights the “double-edged sword” nature of RT: even conventional fractionation can exert significant immunosuppressive effects by depleting the very lymphocyte pools necessary for sustaining systemic antitumor immunity. The ensuing severe lymphopenia likely compromised the durability of the abscopal response and contributed to the failure of subsequent immunotherapy, illustrating that the immunogenic potential of RT is ultimately contingent upon the preservation of systemic immune competence.

The target volume delineation strategy of the RT target area is essential for successful treatment outcomes, necessitating a focus on high-metabolic lesions and multiple lymph node regions that typically exhibit elevated levels of tumor antigens. A meticulous target volume delineation strategy ensures effective targeting of the immune response while minimizing radiation-induced damage to normal tissues (41). Genetic and immune heterogeneity among metastatic lesions is prevalent and can modify the antigenic profile of tumors, thereby influencing the efficacy of immunotherapy (9–15). While the incidence of adverse effects is low with single-site irradiation, multi-site irradiation can expose a broader array of tumor-associated antigens, with certain organs, such as the liver or lungs, being more conducive to eliciting an immune response (16). The tumor-draining lymph node (TDLN) is integral to the orchestration of the AE, serving as a crucial site for T-cell priming and expansion (42–44). Preclinical investigations have revealed that RT not only augments T-cell infiltration in irradiated tumors but also promotes the proliferation of stem-like CD8+ T cells within the TDLN. These T cells subsequently migrate to distant tumor sites, facilitating antitumor responses (45, 46). Disruption of the TDLN, whether through surgical removal or targeted RT, significantly impairs the AE, highlighting its critical role in systemic immune activation (47, 48). Additionally, the TDLN is vital for maintaining the balance of M1/M2 macrophage within the TME, which significantly influences the overall antitumor immune response (49). The size of the radiation field is another important factor affecting the immunogenicity of RT. While larger fields may enhance tumor antigen release and DC recruitment, excessive radiation can lead to immunosuppression by damaging radiosensitive immune effector cells, such as lymphocytes and DCs.

The optimal timing for the administration of RT and ICIs remains a topic of ongoing debate, with no established consensus. The interval between their administration is crucial for maximizing therapeutic efficacy. According to the study by Michael Hettich et al, PD-1 expression peaks within 4 to 6 days post-RT and subsequently declines gradually, suggesting that anti-PD-1 therapy should be initiated concurrently with RT and continued for several days thereafter (50). Recent data from the PACIFIC study indicate a significant improvement in both overall survival and progression-free survival within 14 days following RT, suggesting that the concurrent application of RT and ICIs may elicit a more robust immune response, thereby increasing the likelihood of AE (7). A multicenter retrospective study on brain metastases in NSCLC revealed that patients with an interval of ≤7 days between Stereotactic Body Radiation Therapy (SBRT) and immunotherapy exhibited a longer survival period compared to those with an interval of >7 days (28). In stage IV NSCLC patients, the combination of SBRT with systemic therapy demonstrated comparable safety to sequential therapy, allowing for early systemic treatment without an increase in toxicity (51). These findings suggest that concurrent radioimmunotherapy may be more effective. However, due to limited clinical data, further research is necessary to determine the optimal timing for treatment. The concept of the Immunologically Effective Dose (IED) has been introduced to quantify the intrinsic immunogenicity of radiation therapy schedules. The IED model incorporates parameters such as dose per fraction and inter-fraction time, along with factors related to the local availability of immune effectors, to predict the likelihood of eliciting an abscopal response. This model underscores the importance of customizing radiation field size and dosing regimens to maximize immune activation while minimizing immune suppression, particularly when used in conjunction with ICIs (52, 53).

RT has the capacity to enhance anti-tumor immunity; however, it also exerts immunosuppressive effects that may compromise its long-term effectiveness and contribute to treatment resistance, thereby presenting a “double-edged sword” phenomenon (54–59). A prevalent adverse effect of RT is lymphopenia, as hematopoietic stem cells and peripheral blood lymphocytes exhibit high sensitivity to radiation, resulting in a marked decrease in lymphocyte count and diminished anti-tumor immune function (60). In this particular patient, the use of a small radiation field, despite causing a documented adverse event, also led to significant lymphopenia. After the initiation of CFRT, the ALC showed a gradual initial decline. A marked decline was observed when the cumulative dose reached approximately 40 Gy, with the ALC dropping to a nadir of 0.25×10^9^/L on May 16, 2023, indicating the onset of severe RIL. Although partial recovery was observed following the first course, the second course of RT induced an even more pronounced decline, with the count reaching 0.17×10^9^/L. The persistent and severe depletion of lymphocytes, particularly affecting CD4+ and CD8+ T cells, significantly compromised systemic anti-tumor immune surveillance and response. Despite the transient nature of the AE and the administration of immunotherapy, the patient subsequently experienced rapid systemic progression and ultimately succumbed to the disease, highlighting the critical importance of immune cell count in determining long-term treatment outcomes. An important consideration is the potential synergy with prior immunotherapy. Our patient had received camrelizumab (AiRuiKa^®^) months before RT. Although administered with intervening progression, a residual immunomodulatory “carry-over” effect cannot be entirely ruled out. The observed AE may thus be attributed to the synergy between RT and a pre-primed immune system, rather than RT acting alone.

## 4 Conclusion

In summary, the emergence of immunotherapy has posed challenges to traditional RT. To effectively enhance the occurrence of AE in clinical practice, it is essential to consider various factors, including radiation dose and fractionation, the sequencing of RT and ICIs, the selection of specific ICI drugs, the site of irradiation, the patient’s overall health status, disease stage, and tumor characteristics. This case demonstrates that CFRT can induce an AE even in TSCC, but also vividly illustrates the concomitant risk of severe lymphopenia which may undermine long-term efficacy. Therefore, we emphasize the importance of monitoring absolute lymphocyte counts during such treatments and integrating strategies to mitigate radiation-induced lymphopenia into combination therapy plans to preserve immune competence. This case offers novel clinical evidence and a theoretical basis for the combined treatment of recurrent TSCC, although further investigation into its immunological mechanisms is necessary.

## Data Availability

The original contributions presented in the study are included in the article/supplementary material. Further inquiries can be directed to the corresponding author.
